# Two divergent paths: compression vs. non-compression in deep venous thrombosis and post thrombotic syndrome

**DOI:** 10.1590/1677-5449.006817

**Published:** 2017

**Authors:** Eduardo Simões Da Matta

**Affiliations:** 1 Clínica Pró Circulação™, Xanxerê, SC, Brazil.

**Keywords:** deep vein thrombosis, post-thrombotic syndrome, compression therapy, adjustable velcro compression device, trombose venosa profunda, síndrome pós-trombótica, terapia de compressão, dispositivo de compressão de velcro ajustável

## Abstract

Use of compression therapy to reduce the incidence of postthrombotic syndrome among patients with deep venous thrombosis is a controversial subject and there is no consensus on use of elastic versus inelastic compression, or on the levels and duration of compression. Inelastic devices with a higher static stiffness index, combine relatively small and comfortable pressure at rest with pressure while standing strong enough to restore the “valve mechanism” generated by plantar flexion and dorsiflexion of the foot. Since the static stiffness index is dependent on the rigidity of the compression system and the muscle strength within the bandaged area, improvement of muscle mass with muscle-strengthening programs and endurance training should be encouraged. Therefore, in the acute phase of deep venous thrombosis events, anticoagulation combined with inelastic compression therapy can reduce the extension of the thrombus. Notwithstanding, prospective studies evaluating the effectiveness of inelastic therapy in deep venous thrombosis and post-thrombotic syndrome are needed.

## Resumo

O uso da terapia de compressão para reduzir a incidência de síndrome pós-trombótica em pacientes com trombose venosa profunda apresenta controvérsias como o uso da compressão elástica *versus* inelástica, os níveis e a duração da compressão. Dispositivos inelásticos com índice de rigidez estática combinam uma pressão pequena e confortável em repouso com uma pressão forte o suficiente para restaurar o “mecanismo de válvula” gerado pela flexão plantar e dorsiflexão do pé. Uma vez que o índice de rigidez estática depende da rigidez do sistema de compressão e da força muscular dentro da área enfaixada, a melhoria da massa muscular com programas de fortalecimento e treinamento de resistência deve ser incentivada. Na fase aguda dos eventos de trombose venosa profunda, a anticoagulação acompanhada de terapia de compressão inelástica pode reduzir a extensão do trombo. Assim, são necessários estudos que avaliem a eficácia da terapia inelástica na trombose venosa profunda e na síndrome pós-trombótica.

## INTRODUCTION

The use of compression therapy to prevent the development of postthrombotic syndrome (PTS) in patients with deep vein thrombosis (DVT) remains a source of controversy. According to the latest CHEST Guideline and Expert Panel Report on Antithrombotic Therapy for VTE Disease,^1^ using compression stockings does not contribute to prevention of PTS. A recent randomized placebo-controlled trial concluded that routine use of graduated compression stockings neither lessened PTS nor produced other significant benefits.^2^ However, this conclusion was not itself uncontroversial, and the primary question raised was how a controlled study could measure the therapeutic effect of a procedure that was only sporadically used by most patients.^3^ Furthermore, compression therapy was not initiated immediately after DVT was diagnosed, but after most symptoms had already gone. This may explain the low compliance rates. Nonetheless, as emphasized in the CHEST Guideline, the recommendation focuses on prevention of chronic complications associated with PTS and not on symptomatic treatment.^1^ If the focus is on symptoms, then resolution of pain and edema can be achieved with compression therapy while patients ambulate with concomitant compression.^4^ It is therefore important that compressive therapy and walking should both commence in the acute phase.

We believe that the discussion is all but ended, although there are a number of pending issues that remain to be resolved: use of compression therapy versus its absence; use of elastic versus inelastic compression; levels of elastic compression, and the ideal duration of treatment. When discussing anticoagulation, we often associate subtherapeutic anticoagulation with vitamin K antagonists with development of PTS, in the context of an almost three-fold increase in risk among those with an international normalized ratio below 2.0 for more than 50% of the time.^5^^,^ 6 This problem could be prevented with new oral anticoagulants that achieve more rapid onset and more predictable pharmacokinetics than vitamin K antagonists, ultimately reducing the incidence of PTS.^7^ There is, however, a need to conduct experiments to test these concepts formally. Other important risk factors include age and obesity, the latter doubling the risk of PTS development.^8^

Chronic venous insufficiency (CEAP C3-C6) is a term reserved for advanced chronic venous disorders, as well as for functional abnormalities of the venous system. These conditions lead to various symptoms including edema, skin changes, and venous ulcers,^9^ and may result from a variety of conditions. Damage to the venous valves secondary to the inflammatory process and recanalization of the thrombosed vein decrease venous return to the heart, thereby leading to ambulatory venous hypertension. The size and extent of the initial DVT are equally significant predictors of PTS. The greater the degree of valvular insufficiency, whether primary or secondary, the higher the pressure exerted by the venous liquid column secondary to the reflux in this segment. Deep venous axial reflux at the knee and calf levels is associated with severe venous disease and is independent of the reflux occurring in the external system.^10^ In addition to clinical efforts employed in the treatment of deep venous reflux, there are a number of surgical techniques that could be used, including segmental vein valve transfer and transplantation or venous transposition. These techniques have all been demonstrated to reduce the clinical severity of chronic venous disease.^11^^,^ 12

Furthermore, upon examining the venous physiology, other factors besides valvular reflux can influence venous return. “Vis a Fronte”, or the suction force, originates from the right atrium-thoracoabdominal pump system. Right atrial pressure increases when the right heart pumps poorly, leading to a surge in peripheral venous pressure in the lower extremity.^13^ Also, the thoracoabdominal twin system operates side-by-side with respiration. During the inspiratory peak, the diaphragm descends, and the inferior vena cava is extensively compressed resulting in emptying of the abdominal segment toward the thorax, while distally, a halt in flow occurs secondary to the pressure rise in the inferior vena at the respiratory peak. With expiration, the pressure in the inferior vena cava declines and blood flow is released, allowing for expulsion of blood from the lower extremity.^14^ As a result, all of the following factors result in venous hypertension in the lower limbs: inability of the heart to pump blood out of the right atrium and ventricle into the lungs, failure of the thorax to promote normal respiratory movements, and situations that increase the pressure in the abdominal cavity. These mechanisms also make it easier to understand the association between PTS and obesity.^8^ An increase in abdominal cavity pressure results in a subsequent rise in the venous pressure of the lower limbs, effecting a more superficial respiratory movement and ultimately yielding less efficient thoracic suction. Hence, obesity is yet another factor increasing venous pressure rather than acting through valvular insufficiency. Regarding the venous pumps of the lower extremity, Brizzio,^15^ states that there are seven sectors of aspiration-impulse pumps in the lower extremity, all of which are essential. Their combined work enables blood flow toward the heart.^16^ Studies evaluating lower extremity pump integrity in association with ejection fraction have provided evidence of its connection with muscle strength and joint mobility. The percentage of ejection fraction decreases with a patients’ reduced ability to move their knee or ankle. PTS among elderly patients has been associated with *increased loss of the functional capacity of the lower limbs*.^9^ The elderly frequently present increasing levels of sarcopenia or loss of skeletal muscle mass and function. The progressive decline in muscle mass occurring between the ages of 40 and 80 years is estimated to result in a loss of 30 to 50%,^17^ with associated reductions in functional capacity. It is a significant clinical problem for the elderly, causing a decrease in venous pump function with consequent ambulatory venous hypertension. Elderly patients are therefore more likely to develop signs of Chronic Venous Insufficiency than young or active people. Post-DVT PTS is more likely to occur in a person with impaired ability of the lower extremities as well as among patients with sarcopenia, the elderly, or those with sedentary habits.

Compared with flexible systems, compression therapy systems with a high Static Stiffness Index - SSI (rigid or multi-layer bandage systems) produce higher pressure levels when patients are standing upright and lower pressures when patients are lying down. The best results seem to involve a decrease in ambulatory venous hypertension and an increase in the ejection fraction of the calf pump when an interface pressure of more than 60 mmHg is achieved in the upright position.^18^ Inelastic compression combines a relatively small and comfortable pressure at rest with a standing pressure strong enough to restore the “valve mechanism” generated by plantar flexion and dorsal flexion of the foot. Therefore, inelastic compression is the leading option when it comes to edema, pain, ejection fraction, and restoration of the “valve mechanisms”.^4^^,^ 19 ^,^ 20 There is thus a need to consider this therapeutic option in acute episodes of DVT and subsequent PTS. The challenge with inelastic therapy is the need for healthcare professionals with skills to achieve adequate pressure levels. The short stretch Adjustable Velcro Compression Device (Circaid® Juxta Lite, Medi GmbH, Bayreuth, Germany) can potentially meet this requirement without any previous experience with the device.^21^ In spite of all these allowances, however, there is still no clinical evidence in the literature supporting the application of inelastic therapy in DVT and PTS.

Muscle strength is a prominent factor worth considering when evaluating SSI and Working Pressure Amplitude (WPA). SSI and WPA are not only determined by the rigidity of the applied compression system, but more so by the muscle strength produced inside the bandaged area,^22^ which is one more factor leading to sarcopenia.

## CONCLUSION

The purpose of this review was to propose new ways of thinking about PTS therapy with or without a compression device. In an acute episode of DVT, anticoagulation and compression therapy should be initiated as soon as possible to reduce the extent of the thrombus. At this time, the greater the ejection fraction levels, the higher the rate of venous return, ultimately decreasing the risk of a new thrombus. As a result, inelastic therapy provides increased effectiveness in the initial treatment phase. Results obtained in symptomatic treatment proved to be more efficient with rigid rather than elastic therapy. Concerning the duration of treatment with compression, it is likely unrealistic to believe that the temporary wearing of a compressive method will prevent chronic venous insufficiency secondary to DVT. DVT will lead to a more severe chronic venous insufficiency with thrombus expansion, exacerbating the situation and requiring continuous treatment, as is the case with chronic diseases. Understanding this mechanism allows for a compressive method that reestablishes the damaged valve mechanism with inelastic compression. The short stretch Adjustable Velcro Compression Device (Circaid® Juxta Lite, Medi GmbH, Bayreuth, Germany) should be considered a continuous treatment for this chronic disorder as it does not require previous experience. Another factor worth considering is improvement of muscle mass with proper muscle strengthening programs,^23^ in conjunction with muscle stretching to improve the function of the lower extremity venous muscle pumps. Lymphatic drainage is worth considering as part of the treatment, especially since chronic venous insufficiency is always chronic venous and lymphatic insufficiency.^24^ Aerobic activities as well as muscle endurance training improve venous return and should thus be encouraged, especially since they promote venous and lymphatic return. Control of body weight must also be part of the treatment, as should treatment for any other condition detrimental to the “Vis a Fronte”. Recognition of the disease's progression by the patient and physician, as well as the need for continuous therapy, will guarantee improved treatment compliance by the patient, and a holistic therapeutic approach by the practitioner.
